# Retrograde Labeling of Different Distribution Features of DRG P2X2 and P2X3 Receptors in a Neuropathic Pain Rat Model

**DOI:** 10.1155/2020/9861459

**Published:** 2020-07-22

**Authors:** Lin Chen, Changlong Leng, Qin Ru, Qi Xiong, Mei Zhou, Yuxiang Wu

**Affiliations:** ^1^School of Physical Education, Jianghan University, Wuhan 430056, China; ^2^Wuhan Institutes of Biomedical Sciences, Jianghan University, Wuhan 430056, China

## Abstract

The distributions of P2X subtypes during peripheral neuropathic pain conditions and their differential roles are not fully understood. To explore these characteristics, the lumbosacral dorsal root ganglion (DRG) in the chronic constriction injury (CCI) sciatic nerve rat model was studied. Retrograde trace labeling combined with immunofluorescence technology was applied to analyze the distribution of neuropathic nociceptive P2X1-6 receptors. Our results suggest that Fluoro-Gold (FG) retrograde trace labeling is an efficient method for studying lumbosacral DRG neurons in the CCI rat model, especially when the DRG neurons are divided into small, medium, and large subgroups. We found that neuropathic nociceptive lumbosacral DRG neurons (i.e., FG-positive cells) were significantly increased in medium DRG neurons, while they declined in the large DRG neurons in the CCI group. P2X3 receptors were markedly upregulated in medium while P2X2 receptors were significantly decreased in small FG-positive DRG neurons. There were no significant changes in other P2X receptors (including P2X1, P2X4, P2X5, and P2X6). We anticipate that P2X receptors modulate nociceptive sensitivity primarily through P2X3 subtypes that are upregulated in medium neuropathic nociceptive DRG neurons and/or via the downregulation of P2X2 cells in neuropathic nociceptive small DRG neurons.

## 1. Introduction

Pain is an annoying sensory or experience initiated by a primary lesion or dysfunction in the nervous system [1]. Pain, including peripheral neuropathic pain, represents a major clinical challenge because current treatments provide insufficient pain relief and adverse effects. Systemic drug treatment is often limited in efficacy. The underlying reason is partly due to the plasticity of nociceptive receptors, making it difficult to understand the development of peripheral neuropathic pain. Dissecting the nociceptive receptor plasticity characteristics in sensory neurons allows the design of appropriate receptor-targeted approaches for its treatment [2].

Ion channel activity is considerable during the process of neuropathic pain pathophysiology. Masses of ion channels subtypes are minutely involved in this process, such as the voltage-gated sodium channel (NaV) and the transient receptor potential (TRP) families [3]. P2X receptors also play important roles in neuropathic pain conditions. ATP, a powerful mediator that activates ligand-gated ion channels (P2X receptors), has been reported to be involved in migraine [4] and to participate in pain signaling in the spinal cord [5]. Accumulated evidence shows that the P2X3 purinergic receptor is mainly present in small nociceptive DRG sensory neurons [6], which was later validated using immunohistochemistry [7, 8]. Moreover, some reports have indicated that P2X3 homopolymer activation contributes to acute nociception, while P2X2/3 heteropolymer activation appears to modulate longer-term nociceptive sensitivity in nerve injury [9]. However, other observations have revealed some meaningful phenomena. Some researchers have shown that P2X3 expression during neuropathic pain remains unchanged [10] or decreases after peripheral nerve injury [11]. Even in conditions of spinal nerve ligation, the expression of P2X5 increases while P2X6 decreases [12]. Another study found that P2X5 may be relevant to peripheral pain [13]. Our previous studies using a simple optical density analysis have also demonstrated that P2X1 together with P2X5 receptors are upregulated during neuropathic pain [14].

Despite these reports, data supporting the P2X receptor distribution features of nociceptive DRG neurons are still lacking. The distributions and differential roles of the nociceptive P2X1-6 phenotypes during peripheral neuropathic pain conditions are not fully understood. Therefore, we undertook the present study, combining retrograde tracing together with immunofluorescence methodology, to further explore the nociceptive P2XR distributions of DRG neurons in the sciatic nerve CCI rat model.

## 2. Materials and Methods

### 2.1. Animals

The experimental procedures abided by the Jianghan University Animal Care and Use Committee and complied with National Institutes of Health regulations. Sprague-Dawley (SD) rats (200-220 g, 8 weeks old) were obtained from Wuhan University Center for Animal Experiments (Cat: SCXK, Hupeh 2008-0004). The rats were maintained on a 12-to-12-hour light-dark daily life cycle with access to water and food *ad libitum*.

A randomized 2-animal block allocation was employed to assign animals to one of two experimental groups (*n* = 6 animals/group): control and CCI.

### 2.2. Neuropathic Pain Rat Model and Behavioral Assessment

We developed a neuropathic pain rat model by using unconsolidated constrictive ligatures surrounding the sciatic nerve [15]. Hyperalgesic responses were detected on the third day after surgery and lasted up to two months using this model. Behavioral experiments were performed between 9 and 11 am. During the blinded experiments, the animals were identified by earmarks assigning a number to each mouse, which were announced to the investigator only after completing the experiments and analysis.

Under anesthesia with 2-3% isoflurane in 100% oxygen applied for approximately 1-2 min, maintained through a nose cone to minimize animal suffering during the procedure as described previously, male SD rats were placed in the prone position. The 7 mm sciatic nerve was exposed, and then 4 ligatures (using 4.0 chromic gut) were tied unconsolidated with a spacing of approximately 1 mm near the sciatic trifurcation in the CCI group [15]. Some rats received sham procedures, that is, sciatic exposure without ligation, representing the control group. This kind of control method is beneficial to reduce the influence of extraneous model pain behavior due to receptor expression on the contralateral DRG [16, 17]. Rats were immediately placed on a heating pad (37°C) and administered analgesia (4 mg/kg carprofen subcutaneously) to minimize postprocedural suffering.

**Figure 1:**
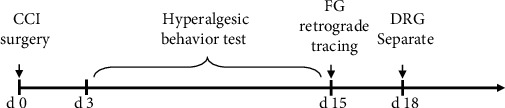
Timeline diagram of the experiment. Twelve rats were randomized into two groups and received CCI or sham surgery procedures on day 0. The pain behavioral tests were performed from day 3 to day 15. FG retrograde trace labeling was performed at day 15. Three days later, the DRG was carefully harvested for further experiments.

On day 3 postsurgery, the spontaneous pain behavior score [18] was rated as in our previous study [14]: 0 score, no obvious behavioral change; 1 score, claw slightly flexed; 2 score, obvious claw buckling and outward rollover; 3 score, lateral, medial margin of claw parts in contact with the glass plate but not bearing; 4 score, animal foot lift, not in contact with the glass plate; and 5 score, in addition to foot lift, licking and biting toe. Thermal hyperalgesia was indicated by testing the paw withdrawal latency (PWL) [19], and the paw withdrawal mechanical threshold (PWMT) was measured to determine the presence of mechanical hyperalgesia [20]. The nociceptive responses were determined using thermal and mechanical hyperalgesia plantar test instruments (Cat: 37370 and Cat: 37450, Ugo Basile, Italy). Concretely, the rats were acclimatized to the apparatus consisting of three individual Perspex boxes on a glass table. A mobile radiant heat source was located under the table and focused on the desired paw, the heat was increased gradually until a withdrawal response was evoked, and the latency of heat needed to cause the withdrawal response was recorded. To prevent tissue damage, an automatic cut-off at 30 sec was set. Rats were placed on a wire mesh floor in clear cylindrical plastic enclosures. Following 20 min of acclimation, a von Frey filament was placed on the plantar surface of the right hind paw, and the force was increased gradually until a withdrawal response was evoked. The amount of force needed to cause the withdrawal response was recorded. A maximum cut-off value of 50 g was used. Each trial was repeated 3 times at ~5 min intervals, and the mean force producing the withdrawal response was determined. These two behavioral tests were performed before ligation surgery and once every other day during the first 14 postoperative days. We only performed behavioral tests and separated the tissue samples on the ipsilateral side. A timeline diagram of the whole experiment is shown in Figure 1.

### 2.3. FG Retrograde Trace Labeling

For retrograde fiber tracing, after the behavioral tests, the sciatic nerves of rats were cut horizontally at midthigh, and then a Silastic tube filled with 7 *μ*l of 2% FG (Cat: FC10001, Fluorochrome LLC, USA) was inserted into the proximal stump. The tube sealing the distal end was attached to the surrounding tissue. Three days later (optimal date for labeling) [21], the corresponding segments (L4-L5) of the DRG, which were verified by tracing the sciatic nerve, were carefully harvested for further study.

### 2.4. Immunofluorescence

All rats were anesthetized using 500 mg/kg sodium pentobarbital administered intraperitoneally and then perfused with 0.9% saline and 4% paraformaldehyde systemically. The L4-L5 segments of the DRG were carefully separated after paraformaldehyde fixation. The paraffined DRG tissues were then cut into 4 *μ*m thick sections. After dewaxing in xylene for two five-minute sessions, graded ethanol (100%, 100%, 95%, 80%, 70%, 50%, and 0%) rehydration treatment was applied every 2 minutes, followed by microwave antigen retrieval in 10 mM sodium citrate for 30 minutes and blocking of endogenous peroxidases in 3% hydrogen for 30 min and 10% normal bovine serum in 0.1 M PBS for 60 min at room temperature. The slices were then incubated with primary antibodies at 4°C overnight. Next, the DRG sections were incubated with Cy3-conjugated secondary antibodies for one hour at room temperature. After nuclear staining with DAPI (RRID: CB73157991, Cat: #S2110, Mounting Medium, antifading (with DAPI), Solarbio Inc., China), the slides were observed and photographs were obtained with a fluorescence microscope system (Olympus, Japan). Cell size measurement was analyzed using cellSens Entry 1.6 software (Olympus, Japan). The primary antibodies were rabbit anti-P2X1-6 (RRID/Cat: AB_2341048, #APR-022; AB_2341051, #APR-025; AB_2341052, #APR-026; AB_2341050, #APR-024; AB_2756757, #APR-027; and AB_2756758, #APR-028, respectively, diluted 1 : 200 with 0.1 M PBS, Alomone Labs, Israel). The second antibody was Cy3-conjugated goat anti-rabbit IgG (Cat: #D110062, diluted 1 : 1000 with 0.1 M PBS, Sangon Biotech (Shanghai) Co., Ltd., China). The negative controls were processed in the same manner except that PBS was used instead of the primary antibody. The specificity test was performed as described in our previous publication [14].

### 2.5. Cell Counting

#### 2.5.1. FG-Positive Cells among Total DRG Neurons

The FG-positive distribution of cells in the DRG, one of the primary endpoints of this study, was detected by counting the FG-positive neurons in this region. DAPI-positive neurons were considered to represent the total number of neurons in the DRG. Three adjacent sections per rat were chosen, and each FG-positive cell was isolated to generate a size distribution. After counting the FG-positive cells, the sections were stained with DAPI and the total cell number was determined to calculate the percentages of FG-positive cells.

The DRG neurons were divided into three subgroups: small, medium, and large (the diameters of the cells in these subgroups were 10-20 *μ*m, 20-40 *μ*m, and greater than 40 *μ*m, respectively) [22, 23]. The same method was utilized to calculate FG-positive percentages for each of the three subgroups of different-sized DRG neurons.

#### 2.5.2. P2X1-6-Positive Cells among Total DRG Neurons

The P2X1-6 receptor distributions of DRG as other primary endpoints of this study were detected by counting the numbers of P2X1-6-positive neurons throughout the DRG. Three randomly selected sections per rat were captured, and each P2X1-6-positive cell was isolated to generate a size distribution. After identifying the P2X1-6-positive profiles, the cell numbers were determined and the P2X-positive percentages were finally calculated.

#### 2.5.3. P2X1-6-Positive Cells among FG-Positive Cells

Counts were determined for both FG-positive and P2X1-6-positive cells. The percentages of P2X1-6-positive among the total FG-positive cells were finally calculated.

The total number of FG-positive DRG neurons was then divided into small, medium, and large cell subgroups. The same method was applied to the different-sized FG-positive cells to calculate the P2X1-6-positive percentages in these three subgroups.

### 2.6. Data Analysis

Behavioral data are presented as means ± SEM, with *n* equal to the number of animals studied. The behavioral tests were evaluated by two-way repeated-measures analysis of variance (ANOVA). Cell count data were collected from sections showing the nucleus (stained with DAPI), which represented the maximum observable diameter of the cell. The average ratios of P2X1-6 and/or FG-positive cells were compared across ages using one-way ANOVA followed by a post hoc Tukey's test. *P* values < 0.05 were considered statistically significant. IBM SPSS Statistics 19 was used for all statistical analyses.

## 3. Results

### 3.1. Establishment of the Neuropathic Pain Rat Model and Behavioral Assessment

**Figure 2:**
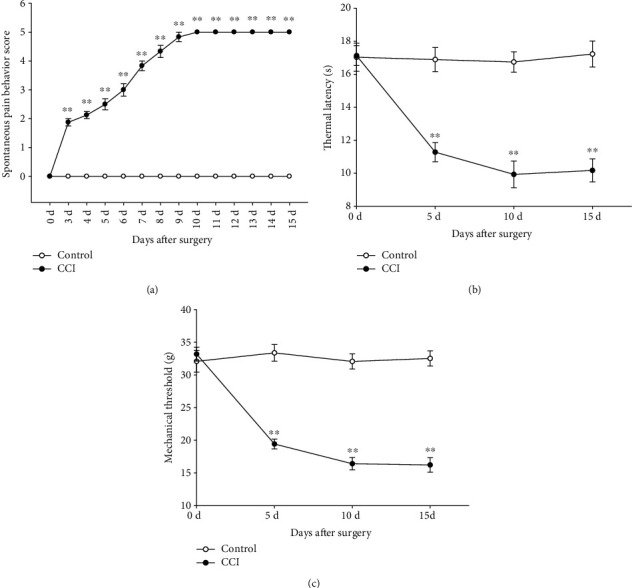
Rat spontaneous pain behavior rating score (a), thermal hyperalgesia withdrawal latency (b), and paw withdrawal mechanical threshold (c) after CCI surgery. The score increased, and latencies decreased from the third day and reached a peak on the 9^th^ or 10^th^ day. These indicators could be sustained for 15 days until the material was obtained from the rats. There was an especially significant difference between the control and CCI groups. ^∗∗^*P* < 0.01, compared with the control group, *n* = 6.

Hyperalgesia was observed on the 2nd postoperative day and lasted up to two months as described previously [15, 18]. The spontaneous pain behavior score (Figure 2(a)), thermal hyperalgesia withdrawal latency (PWL) (Figure 2(b)), and paw withdrawal mechanical threshold (PWMT) (Figure 2(c)) were determined. The results revealed a statistically significant difference between the control and CCI groups (*P* < 0.01), which indicated that the hyperalgesia model was successfully established.

### 3.2. Expression and Distribution of FG-Positive Cells

**Figure 3:**
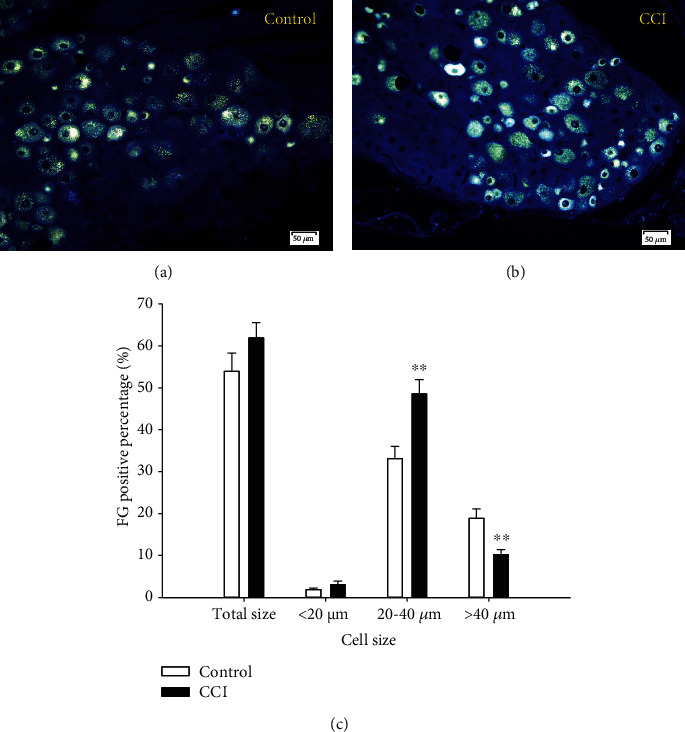
Expression and size distribution of FG-positive cells among total DRG neurons between the control and CCI groups. (a, b) Show the retrograde trace-labeled FG-positive fluorescence images of the DRG (200x, scale is 50 *μ*m). (c) The percentages of expression and size distribution of FG-positive cells to total DRG neurons between the control and CCI groups. The differences were especially significant between the control and CCI groups in medium and large cells, respectively. ^∗∗^*P* < 0.01, compared with the control group, *n* = 6.

The FG-positive cells constituted 53.9% of the control group and rose to 61.9% in the CCI group compared with the total DRG neurons (Figure 3), but there was no obvious difference between them. When the total number of DRG neurons was divided into three subgroups: small, medium, and large cells, the FG-positive cell percentages in these different subgroups showed significant variation. They comprised 33.1% of the medium DRG neurons in the control group but rose to 48.6% in the CCI group (*P* < 0.05). Simultaneously, the FG-positive cell percentage among large DRG neurons was 18.9% in the control group but dropped to 10.2% (*P* < 0.05) in the CCI group. The percentages were quite low (1.9% and 3%) with no remarkable differences between them in small DRG neurons.

### 3.3. Expression and Size Distribution of P2X1-6 Receptors

**Figure 4:**
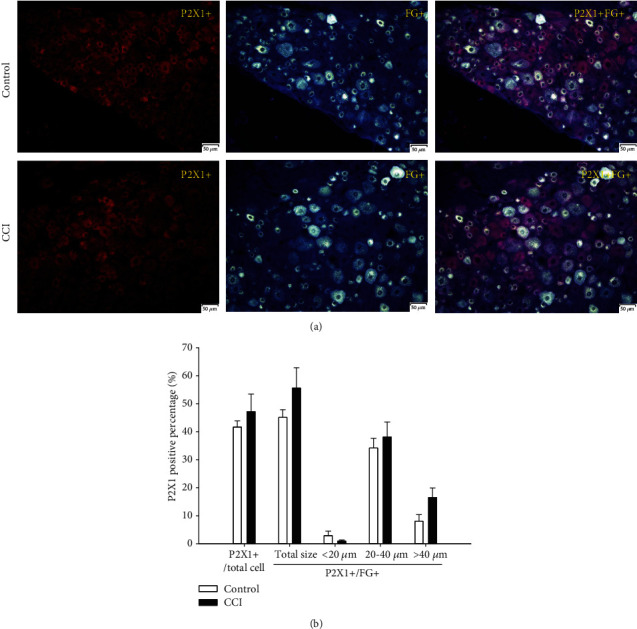
Expression and size distribution of P2X1-positive cells to total and FG-positive DRG neurons between the control and CCI groups. (a) P2X1-positive fluorescence images of total and FG-positive DRG between the control and CCI groups (200x, scale is 50 *μ*m). (b) The percentages of the expression and size distribution of P2X1-positive cells to total and FG-positive DRG neurons between the control and CCI groups. There was no significant difference between the control and CCI groups, *n* = 6.

**Figure 5:**
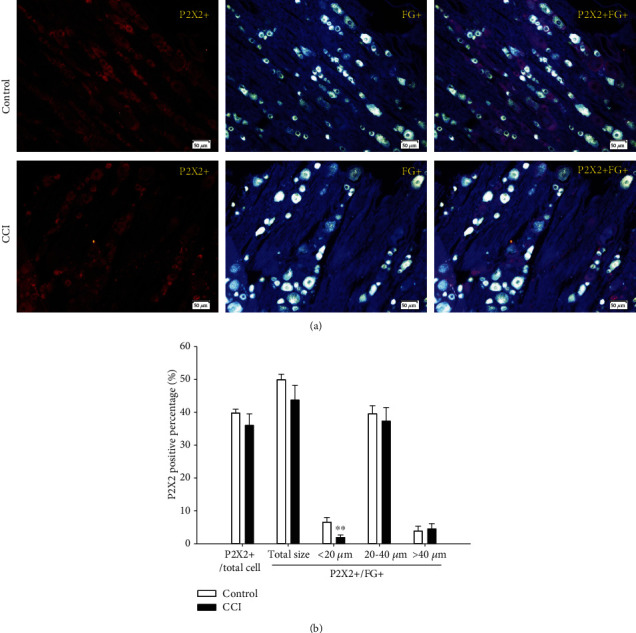
Expression and size distribution of P2X2-positive cells among total and FG-positive DRG neurons between the control and CCI groups. (a) P2X2-positive fluorescence images of total and FG-positive DRG between the control and CCI groups (200x, scale is 50 *μ*m). (b) The percentages of the expression and size distribution of P2X2-positive cells to total and FG-positive DRG neurons between the control and CCI groups. There was an especially significant difference between the control and CCI groups for small cells. ^∗∗^*P* < 0.01, compared with the control group, *n* = 6.

**Figure 6:**
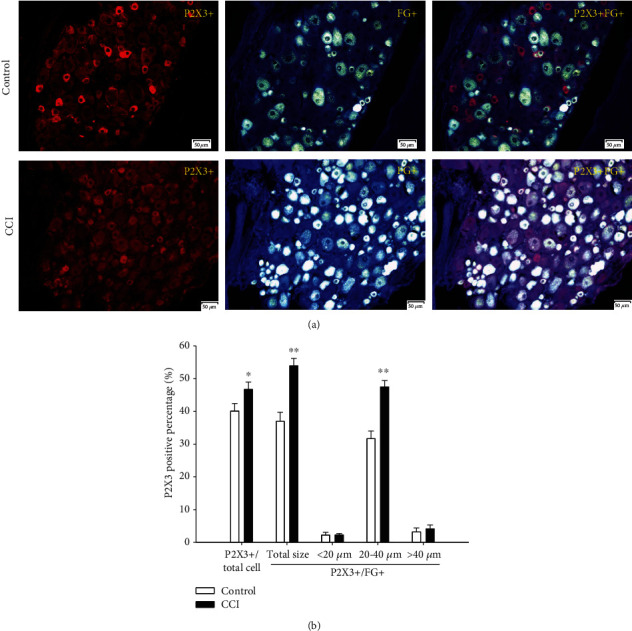
Expression and size distribution of P2X3-positive cells among total and FG-positive DRG neurons between the control and CCI groups. (a) P2X3-positive fluorescence images for total and FG-positive DRG between the control and CCI groups (200x, scale is 50 *μ*m). (b) The percentages of expression and size distribution of P2X3-positive cells among total and FG-positive DRG neurons between the control and CCI groups. Especially significant differences were determined between the control and CCI groups for the total and FG-positive DRG neurons, especially for medium cells. ^∗^*P* < 0.05, ^∗∗^*P* < 0.01, compared with the control group, *n* = 6.

**Figure 7:**
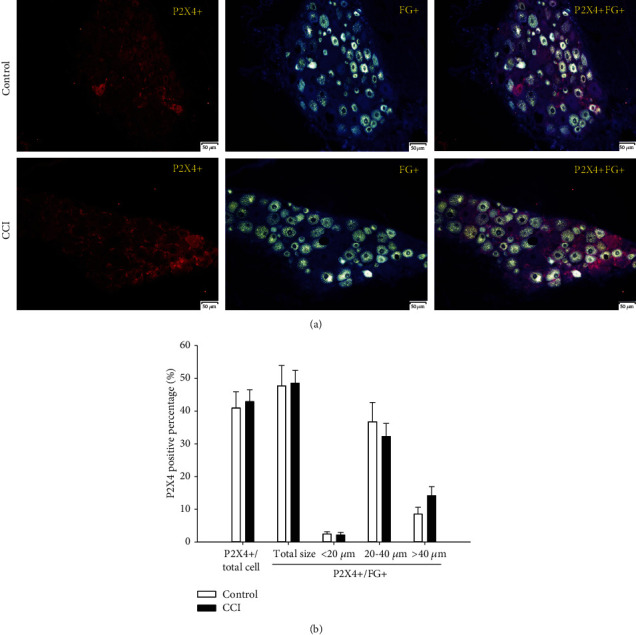
Expression and size distribution of P2X4-positive cells among total and FG-positive DRG neurons between the control and CCI groups. (a) P2X4-positive fluorescence images of total and FG-positive DRG between the control and CCI groups (200x, scale is 50 *μ*m). (b) The percentages of expression and size distribution of P2X4-positive cells among total and FG-positive DRG neurons between the control and CCI groups. There were no significant differences between the control and CCI groups, *n* = 6.

**Figure 8:**
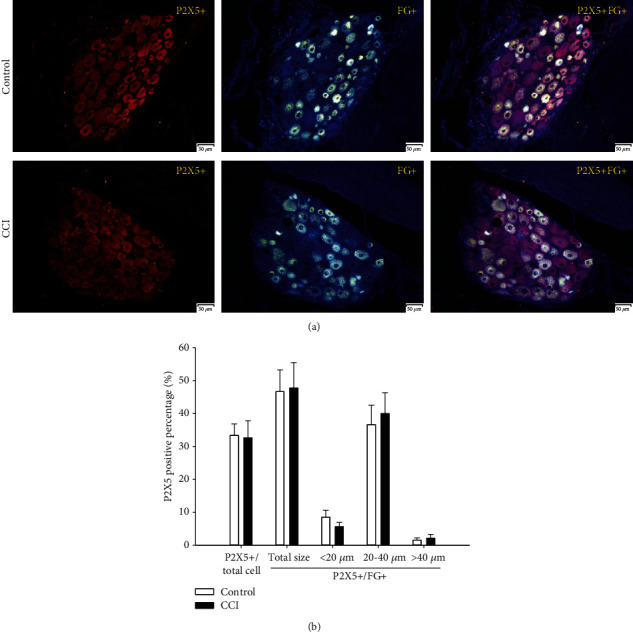
Expression and size distribution of P2X5-positive cells among total and FG-positive DRG neurons between the control and CCI groups. (a) P2X5-positive fluorescence images among total and FG-positive DRG between the control and CCI groups (200x, scale is 50 *μ*m). (b) The percentages of expression and size distribution of P2X5-positive cells among total and FG-positive DRG neurons between the control and CCI groups. There were no significant differences between the control and CCI groups, *n* = 6.

**Figure 9:**
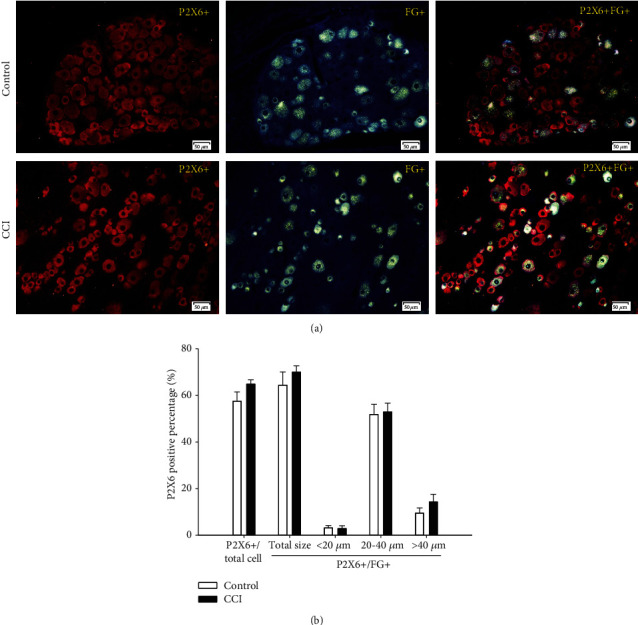
Expression and size distribution of P2X6-positive cells among total and FG-positive DRG neurons between the control and CCI groups. (a) P2X6-positive fluorescence images among total and FG-positive DRG between the control and CCI groups (200x, scale is 50 *μ*m). (b) The percentages of expression and size distribution of P2X6-positive cells among total and FG-positive DRG neurons between the control and CCI groups. There were no significant differences between the control and CCI groups, *n* = 6.

P2X1-6 receptors were expressed in L4-L5 rat DRG (Figures 4–9). Immunoreactive cells were counted to calculate the positive neuron proportions as described in the previous section.

#### 3.3.1. P2X1-Positive Percentages

As shown in Figure 4, the P2X1-positive cell percentage among total DRG neurons was 41.7% in the control group and 47.1% in the CCI group. The P2X1-positive cell percentage among FG-positive DRG neurons was 45.1% in the control group and 55.6% in the CCI group. Although the CCI group percentage was superior to the control group, there was no meaningful difference between these groups regardless of whether the comparison was to total or FG-positive cells. Furthermore, when the FG-positive DRG neurons were divided into different-sized subgroups, the P2X1-positive cell percentage in the medium- and large-sized subgroups showed the same tendency without a significant difference. The P2X1-positive cell percentage in the medium and large DRG neurons was 34.2% and 8% in the control group but rose to 38.1% and 16.5% in the CCI group, respectively. Conversely, the expression of P2X1-positive cells was quite low (2.8% and 0.5%) in the small DRG neurons. Although the P2X1-positive level declined in the CCI group compared with the control group among these small cells, no remarkable differences were identified between them.

#### 3.3.2. P2X2-Positive Percentages

As shown in Figure 5, the P2X2-positive cell percentage among total DRG neurons was 39.8% in the control groups and 36% in the CCI group, with no significant difference. Similarly, the P2X2-positive cell percentage among FG-positive cells was 49.9% in the control groups and 43.7% in the CCI group, again with no significant difference. However, when the FG-positive DRG neurons were divided into subgroups of different size, the P2X2-positive cell percentage decreased significantly in the CCI group compared with the control rats for small DRG neurons (from 6.6% to 1.9%, *P* < 0.01). The percentage of P2X2-positive cells among medium DRG neurons was 39.5% in the control group but declined to 37.2% in the CCI group, with no significant difference. In contrast, the percentage of large DRG cells was 3.8% in the control group and rose slightly to 4.6% in the CCI group, but again without a significant difference.

#### 3.3.3. P2X3-Positive Percentages

As shown in Figure 6, the P2X3-positive cell percentage among total DRG neurons was 40.1% in the control group and rose significantly to 46.7% in the CCI group (*P* < 0.05). Even more strikingly, when compared to the FG-positive DRG neurons, the P2X3-positive cell percentage was 36.9% in the control group and highly significantly upregulated to 53.9% in the CCI group (*P* < 0.01). When the FG-positive DRG neurons were divided into small, medium, and large cell subgroups, the P2X3-positive cell percentage in these different subgroups also increased in the CCI group. The major contribution of P2X3-positive cells was determined in the medium DRG neurons, representing 31.6% in the control group but soaring to 47.5% in the CCI group (*P* < 0.01). Moreover, the percentages of 2.1% and 3.1% in the control group showed slight increases to 2.3% and 4.1% in the large and small DRG-neurons in the CCI group, respectively, but without a marked difference.

#### 3.3.4. P2X4-Positive Percentages

As shown in Figure 7, the P2X4-positive cell percentage among total DRG neurons was 41% in the control group and rose slightly to 42.9% in the CCI group. The P2X4-positive cell percentage among FG-positive DRG neurons also rose from 47.7% in the control group to 48.5% in the CCI group, without significant difference. When the FG-positive DRG neurons were divided into three subgroups, the P2X4-positive cell percentage in small and medium DRG neurons was 2.4% and 36.7% in the control group but dropped to 2.2% and 32.2% in the CCI group, respectively, without significant differences. However, the P2X4-positive cell percentage among large DRG neurons was 8.5% in the control group and 14.2% in the CCI group, but without a marked difference.

#### 3.3.5. P2X5-Positive Percentages

As shown in Figure 8, the P2X5-positive cell percentage of total DRG neurons was 33.3% in the control group and 32.7% in the CCI group. The P2X5-positive cell percentage among FG-positive DRG neurons was 46.7% in the control group and 47.7% in the CCI group. There was no obvious difference between these two groups. The P2X5-positive cell percentage of these different subgroups showed several variations. The P2X5-positive cell percentage among medium DRG neurons was 36.6% in the control group but rose to 40% in the CCI group. However, the P2X5-positive cell percentage of small DRG neurons was 8.6% in the control group but dropped to 5.6% in the CCI group. The expression of P2X5-positive cells was quite low (1.5% and 2.1% in the control and CCI groups, respectively) in large DRG neurons. Similarly, there was no remarkable difference between them.

#### 3.3.6. P2X6-Positive Percentages

As shown in Figure 9, the P2X6-positive cell percentage of total DRG neurons was 57.5% in the control group and increased to 64.9% in the CCI group, while the P2X6-positive cell percentage of FG-positive DRG neurons was 64.4% in the control group and rose to 70% in the CCI group. However, there were no significant differences between the two groups. Furthermore, when the FG-positive DRG neurons were divided into small, medium, and large subgroups, the FG-positive cell percentage among medium and large DRG neurons in the control group was 51.9% and 9.5%, respectively, and rose to 52.9% and 14.3% in the CCI group, although this increase was not significant. The FG-positive cell percentage among small DRG neurons was 3% in the control and 2.8% in the CCI group, again with no obvious differences.

## 4. Discussion

Pain is a complicated sensory process and is physiologically adaptive when facing dangerous stimuli present in the organism's surroundings. Neuropathic pain is induced by activation of A-*δ* and/or C-nociceptors when responding to a noxious stimulus. It results in the release of nociceptive materials to activate peripheral nerve effectors and increase the excitability of superior neurons [24]. Numerous receptors, neurotransmitters, and second messengers together with other signaling molecules are involved in the processing of pain [24, 25]. The complexity and diversity of pain receptor plasticity and sensory neuron sensitization and the involvement of neurotransmitters render it difficult to comprehend pain development progression [2].

To date, our understanding of the spatial/temporal mechanisms of pain mediators from complex cells and tissues is far from completely clear and is further complicated by the receptor plasticity within a cell type context [26]. A large amount of evidence supports the nociceptive receptor subtypes involved in neuropathic pain conditions, obtained (such as NaV, TRP, and P2X) after cloning and characterization of these receptors from sensory nerves. However, more detailed research is needed, in particular during peripheral neuropathic pain conditions.

Retrograde labeling of DRG neurons is based on selective neuronal uptake at terminals and retrograde transport of the neurotransmitter [27]. Moreover, fluorescent tracers can easily be associated with immunocytological methods to characterize the desired labeled cells [28]. A newer generation of retrograde fluorescent tracers such as Fluoro-Gold [29] is extremely resistant to photobleaching. The intense and bleach-resistant labeling has contributed to raising FG to the level of “gold standard” for fluorescent retrograde labeling in rodents, particularly for labeling multiple targets in combination with other tracers. In a living animal, FG may resist metabolic breakdown up to a year postinjection. It survives rigorous immunofluorescence procedures and can be used to mark neuronal cell bodies [30, 31]. Therefore, FG can be considered today's flagship of the retrograde fluorescence tracer repertoire [32]. Among several classic tools that are widely used for retrograde neurodissection tracing purposes, Fluoro-Gold appears to be the best example [33].

In our present research, FG was used as a retrograde tracer, which efficiently labels neurons [34, 35]. It was used together with immunofluorescence methods to explore the P2XR expression profile in the activated lumbosacral DRG in CCI model rats. After CCI surgery, nociceptive pain behaviors were assessed by both spontaneous and evoked stimulation (thermal, mechanical). The spontaneous pain behavior score (Figure 2(a)), PWL (Figure 2(b)), and PWMT (Figure 2(c)) were observed. The results showed a statistically obvious difference between the control and CCI groups (*P* < 0.01), which suggested that the neuropathic pain model was successfully established.

Next, the percentage of FG-positive cells was calculated (shown in Figure 3), which ranged from 53.9% in the control group to 61.9% in the CCI group, with no significant difference when compared to the total DRG neurons. When the total DRG neurons were divided into small, medium, and large cells, the FG-positive cell percentage showed significant variation among these different subgroups. Among medium DRG neurons, the percentage rose significantly in the CCI group (*P* < 0.05), while among large DRG neurons, it clearly dropped in the CCI group (*P* < 0.05). The expression of FG-positive cells was quite low among small DRG neurons, with no marked difference between the groups. These results indicated that FG retrograde trace labeling is an efficient method to obtain more details in activated lumbosacral DRG neurons in the sciatic nerve CCI rat model.

To obtain greater detail, the total DRG neurons were further divided into three subgroups to calculate the positive percentages: small, medium, and large cells (the diameters of these subgroup cells ranged from approximately 10-20 *μ*m, 20-40 *μ*m, and more than 40 *μ*m, respectively) based on previous research results [8, 23] and our [22] previous findings: the recorded slow, intermediate (or mixed), and fast types of ATP-activated current correlated well with cell size (cell diameter: 21.2 ± 3.0, 38.7 ± 3.9, and 45.8 ± 5.9 *μ*m in sequence).

P2X receptors belong to the cation channel family with gates controlled by ATP, and they play important roles in the peripheral and central nervous systems [36–38]. P2X receptors of sensory neuron participate in numerous types of sensory modulation, such as pain- and stretch-sensing [8]. Many reports have characterized P2X receptors on sensory neurons, such as those in the DRG and trigeminal regions, among others [39]. Previous researchers have used whole-cell patch clamp recordings to study the expression of P2X receptor phenotypes and distribution patterns in DRG neurons [40]. The results revealed three types of P2X currents: fast, slow, and mixed. Each of these P2X receptor phenotypes had a distinct distribution pattern among DRG neurons. The fast P2X currents were predominantly expressed in small-diameter DRG neurons. The slow P2X currents were expressed in both small and medium DRG neuron. The mixed P2X currents were also expressed in both small- and medium-sized DRG neurons. The desensitization time domain of P2X indicates the response during the current elicited by ATP. In some P2X receptors, the desensitization time domain decline occurs in milliseconds (fast desensitization: P2X1, P2X3); in others, it occurs 100-1,000 times more slowly (slow desensitization: P2X2, P2X4) [41]. P2X receptors can also form multiple hypotypes [6, 7, 42, 43]. Thus, the relationships among P2X receptor phenotypes and cell sizes are more complicated. Different P2X receptors may be involved in both nociceptive and nonnociceptive functions. Clarifying the different roles of P2X expression in DRG neurons of different size is of great importance to understand the peripheral mechanism of neuropathic pain and to explore new analgesic targets.

All P2X hypotypes are located on trigeminal neurons, and P2X3 shows the highest expression [44]. Several more reports support that the nociceptive actions caused by ATP are mediated in part by activated P2X3 receptors, which are restricted to nociceptive primary sensory neurons and present in 1/3 of L4/5 DRG [45]. P2X3 and P2X2/3 are mainly expressed on small nociceptive neurons [45]. Moreover, some reports suggested that the activation of P2X3 induces acute nociception, while activation of P2X2/3 appears to modulate longer-term nociceptive sensitivity in nerve injury [9]. The P2X3 receptor is involved in acute pain, chronic neuropathic pain, inflammatory pain, and even cancer pain [46], and the sensitization of P2X3 is prominently related to neuropathic pain [47]. However, some studies have shown no change in P2X3 expression during neuropathic pain [10] or a decrease in expression [11, 45].

The present P2X3 expression profile results consolidated and demonstrated that the major contributors to P2X3 upregulation in the CCI group were the P2X3-positive cells among medium-sized DRG neurons. As can be observed in Figure 6(b), the percentage of P2X3-positive cells among total DRG neurons rose significantly in the CCI group (*P* < 0.05). Even more strikingly, when compared with FG-positive DRG neurons, the percentage of P2X3-positive cells was highly significantly upregulated in the CCI group (*P* < 0.01). When the FG-positive DRG neurons were divided into small, medium, and large cell subgroups, the percentages of P2X3-positive cell in these different subgroups also increased in the CCI group. The percentages of P2X3-positive cells among medium DRG neurons soared in the CCI group (*P* < 0.01). Moreover, the percentages of P2X3-positive cells rose slightly among large and small DRG neurons in the CCI group, but without a marked difference.

It has been previously reported that the P2X2/3 heteromultimer is expressed on small nociceptive neurons and is particularly prominent in the nodose ganglion [45]. Some researchers consider P2X2/3 activation to regulate longer-term nociceptive sensitivity during nerve injury [9]. The expression of P2X2 as well as P2X3 receptors obviously increased in the neuropathic pain model [48, 49]. Regardless of these findings, we observed, as shown in Figure 5(b), that the percentage of P2X2-positive cells among total DRG neurons decreased in the CCI group, though without a significant difference. Similarly, the percentage of P2X2-positive cells among FG-positive cells decreased in a nonsignificant manner in the CCI group. However, when the FG-positive DRG neurons were divided into subgroups of different size, the P2X2-positive cell percentage significantly decreased among small-sized DRG neurons in the CCI group (*P* < 0.01). The percentage among medium DRG neurons declined while increased slightly among large DRG neurons in the CCI group, both without a significant difference. These findings might indicate that the heteromeric P2X2/3 receptor involves nociceptive sensitivity through P2X3 subtypes that are upregulated in medium neuropathic nociceptive DRG neurons and/or the decline in P2X2 cells among small neuropathic nociceptive DRG neurons.

Regarding P2X1, P2X4, P2X5, and P2X6, some experiments have shown that the expression of P2X3 or P2X6 decreases but P2X5 increases [12]. Other studies have suggested that P2X5 is also potentially related to peripheral pain [13]. Even our previous neuropathic pain studies have pointed out that in addition to P2X2 and/or P2X3, P2X1 and P2X5 also increase based on a simple optical density analysis [14]. P2X2, P2X4, and P2X6 receptors of DRG also pass nociceptive information during the pain process [37]. The mechanical hyperalgesia is reduced in P2X4-deficient mice during peripheral nerve injury [50].

The present study showed that the expression of the present receptor subtypes was widespread in the DRG during neuropathic pain, in accordance with some previous studies [7, 13, 51, 52]. Generally, these receptors showed the same slight upregulation trends without a significant difference. Specifically, the P2X1-positive cell percentage among medium and large DRG neurons slightly rose but decreased among small DRG neurons in the CCI group. The P2X4-positive cell percentage among small and medium DRG neurons dropped in the CCI group without a significant difference. Conversely, the percentage among large DRG neurons rose in the CCI group. The P2X5-positive cell percentage among medium DRG neurons rose to 40% but among small DRG neurons dropped to 5.6% in the CCI group. The expression of P2X5-positive cells was quite low in large DRG neurons. The P2X6-positive cell percentage among medium and large DRG neurons rose in the CCI group without significant differences. The FG-positive cell percentage among small DRG neurons was the same in the control compared with the CCI group.

We also noticed that the general trend of the experimental results was the same during different stages of research, but some details may have differed, which may have been due to different research focuses in each stage, as well as individual differences, operational details, and experimental error among samples in each stage. Initially, we used immunohistochemistry alone to evaluate the expression and distribution of P2X receptor subtypes in L4-L5 segment DRG in the neuropathologic pain state. Preliminary findings revealed that, excluding P2X2 and P2X3, the expression levels of P2X1 and P2X5 receptors increased during neuropathic pain [14]. To further clarify which P2X receptor subtypes characteristically changed in pain-activated DRG, retrograde trace labeling combined with immunofluorescence technology was applied to analyze the distribution of P2X1-6 receptors among the pain-activated L4-L5 DRG. With respect to the “total size” DRG, we found no significant difference in the proportion of FG-labeled DRG neurons between the sham and CCI groups. The percentages of P2X1and P2X2 FG-labeled DRG neurons were not significantly different between the sham and CCI groups. The percentages of P2X3 and P2X6 FG-labeled DRG neurons significantly increased in the CCI compared with the sham group. By contrast, the percentages of P2X4 FG-labeled DRG neurons significantly decreased in the CCI compared with the sham group. P2X5-positive FG-labeled neurons were not detected in the CCI and sham groups [53]. We repeated the same retrograde trace labeling combined with immunofluorescence experiment on the CCI rats L4-L5 DRG to explore the distribution characteristics of neuropathic nociceptive P2X1-6 receptors of the “different three sizes” of DRG. The main finding was that FG-positive cells were significantly increased among medium DRG neurons while declining in the large DRG neurons in the CCI group, although there was no significant difference in the proportion of FG-labeled DRG neurons between the sham and CCI groups. P2X3 receptors were dramatically upregulated among medium-sized FG-positive DRG neurons but significantly decreased among small FG-positive DRG neurons. Although there were no significant difference in other P2X receptors (including P2X1, P2X4, P2X5, and P2X6), the general trends were consistent with the previous experiment, such that P2X1 increased among all DRG sizes, P2X4 decreased in medium DRG, P2X5 increased in all and in medium DRG, and P2X6 increased in all and in large DRG, but without significant differences.

Furthermore, neuronal signal pathways, satellite cells, Schwann cells of the DRG, and even microglia and astrocytes are all involved in the development of neuropathic pain [54]. P2X1-7 is expressed on astrocytes, P2X1 is expressed on oligodendrocytes, and P2X4 and P2X7 are expressed on microglia [39]. Microglial P2X4 and P2X7 receptors have been reported to be involved in peripheral nerve injury-induced neuropathic and inflammatory pain [55, 56]. After peripheral injury or during chronic pain, abnormal crosstalk between neuronal and nonneuronal cells at the ganglion level has an important role in activation of the local vascular and immune system [57, 58]. In this situation, the recruitment of new receptors occurs at the cell membrane level (neurons, glia, and inflammatory cells), which are involved in hyperalgesia and allodynia [59, 60]. Extracellular matrix-integrin signaling, altered glial communication, and the role of pannexin hemichannels at the ganglion level all have key roles in pain progression [61]. Extracellular ATP is one of the most powerful molecules influencing the neuronal sensitization process and inflammation [62] at the peripheral and central levels. The mechanistic link between ATP and its receptors at the cell membrane level in those complex neural-immune contexts should be further investigated.

## 5. Conclusions

In the present research, FG retrograde labeling combined with an immunofluorescence method was utilized to explore the P2XR expression profile in neuropathic nociceptive lumbosacral DRG neurons in the CCI rat model. Our results suggest that FG retrograde trace labeling is an efficient method to obtain more details about neuropathic nociceptive lumbosacral DRG neurons in the CCI rat model, especially when the DRG neurons are divided into three subgroups. We found that the neuropathic nociceptive lumbosacral DRG neurons (that is, the FG-positive cells) significantly increased among medium-sized while declining among the large-sized DRG neurons in the CCI group. P2X3 receptors were dramatically upregulated among medium while P2X2 receptors were significantly decreased among small FG-positive DRG neurons. There were no significant changes in other P2X receptors (include P2X1, P2X4, P2X5, and P2X6). We anticipate that P2X receptors appear to modulate nociceptive sensitivity primarily through the upregulated P2X3 subtypes in medium neuropathic nociceptive DRG neurons and/or the downregulated P2X2 cells among neuropathic nociceptive small DRG neurons.

## Data Availability

The datasets generated and/or analyzed during the current study are available from the corresponding author on reasonable request.
